# MELD: A multilingual ethnic dataset of Chakma, Garo, and Marma in Bengali script with English and standard Bengali translation

**DOI:** 10.1016/j.dib.2025.111745

**Published:** 2025-06-04

**Authors:** Mehraj Hossain Mahi, Anzir Rahman Khan, Mobashsher Hasan Anik, Sheak Rashed Haider Noori, Arif Mahmud, Mayen Uddin Mojumdar

**Affiliations:** Multidisciplinary Action Research Lab, Department of Computer Science and Engineering, Daffodil International University, Birulia, Dhaka 1216, Bangladesh

**Keywords:** Ethnic language, Transliteration, Natural language processing, Ethnic language identification, Machine translation

## Abstract

There are thousands of ethnic groups in the world contributing to the rich linguistic and cultural diversity of people. However, in digital resources and research, the majority of these languages, including more than 30 ethnic languages spoken in Bangladesh remain severely underrepresented. There is little to no work addressing the preservation, translation, or computational processing of these languages, despite their unique linguistic structures and speaker population. In order to highlight the difficulties faced by low-resource and endangered languages worldwide, this dataset focuses on the three leading ethnic languages, Chakma, Garo, and Marma along with their corresponding Bengali and English translations. People from different ethnic groups use Bengali alphabets to write their own language on social media platforms like Facebook and Twitter, as well as in their daily lives. Due to significant linguistic variances, even when ethnic native speakers use the Bengali script to write their languages, the resulting text is unintelligible to Standard Bengali speakers. Moreover, the lack of translation systems and language identification tools indicate the digital exclusion of these communities. This dataset addresses these gaps by documenting sentence-level linguistic samples in Chakma, Garo, and Marma through transliteration, where the phonetics of each language are represented using Bengali script. It also provides meaning-based translations in both Standard Bengali and English, rather than literal word-for-word mappings, to preserve the intended meaning of the original sentences. By documenting linguistic samples in Chakma, Garo, and Marma through a transliteration process, this dataset is a critical resource for advancing Natural Language Processing (NLP) and cultural preservation for worldwide low-resource ethnic languages.

Specifications Table

**Table:** 

Subject	Computer Sciences
Specific subject area	Underrepresented Languages, Ethnic Linguistics, Low-Resource Languages
Type of data	Table (text/string)
Data collection	Data were collected through interviews with native speakers and written contributions, followed by manual transliteration into the Bengali script. Seven university students—three Chakma, two Garo, and two Marma—volunteered for this initiative, motivated by their commitment to preserving and promoting their mother tongues. Fluent in both their respective ethnic languages and Standard Bengali, these students acted as mediators, transcribing and transliterating the spoken data obtained directly from native speakers in their respective regions.
Data source location	These Data were collected from the following areas in Bangladesh: Khagrachari Sadar, Khagrachari (Latitude: 23° 7′ 24.7728′′ N, Longitude: 91° 58′ 57.414′′ E), Guimara, Khagrachari (Latitude 22° 58′ 14.7972′′N, Longitude: 91° 52′ 30.3924′′ E), Mymensingh Sadar, Mymensingh (Latitude: 24° 44′ 51.0072′′N, Longitude: 90° 24′ 18.9828′′ E)
Data accessibility	Repository name: Mendeley Data Data identification number: 10.17632/dy5dyfygbp.4 Direct URL to data: https://data.mendeley.com/datasets/dy5dyfygbp/4

## 1 Value of the Data

• MELD (Multilingual Ethnic Language Dataset) is the first-of-its-kind transliterated multilingual dataset for Chakma, Garo, and Marma languages, offering sentence-level translations into both Standard Bengali and English. It serves as a rare and invaluable resource, bridging the gap in digital tools for Chakma, Garo, and Marma languages by providing sentence-level translations into both Standard Bengali and English, preserving the original meaning rather than relying on literal word-for-word translation.
• It provides linguists and NLP researchers with a valuable foundation for exploring and analyzing underrepresented languages. The inclusion of Standard Bengali alongside Chakma, Garo, and Marma serves as a benchmark for linguistic comparisons, facilitates translation research, and opens avenues for deeper exploration into the unique characteristics of these ethnic languages.
• To address the lack of digital resources for these ethnic languages, researchers can use MELD to develop cutting-edge technologies for language identification, machine translation, and cultural preservation.
• Currently, most state-of-the-art generative AI models lack knowledge and understanding of these low-resource languages, making MELD a foundational step toward bringing Chakma, Garo, and Marma into the realm of modern AI applications. The dataset paves the way for building translation systems, identification tools, and multilingual chatbots that can understand and generate text in these languages.
• MELD promotes inclusivity and diversity in language technology while highlighting the value of conserving and respecting the traditions of low-resource languages including Chakma, Garo, and Marma through its support of linguistic diversity research. It can also be used for educational purposes and encourage researches to work on underrepresented languages.

## 2 Background

Ethnic languages represent a rich cultural heritage but remain significantly underexplored in digitalization and research [1]. Presently, language extinct or loss is a widespread occurrence in many parts of the world, and Bangladesh is no exception. It has been discovered that Bangladesh's sociolinguistic situation puts several of its indigenous or ethnic languages at risk of “Language Loss” [2]. Bengali, as the national language, dominates formal domains and significantly influences every spheres, potentially endangering the use of ethnic languages in future generations [3]. While over 54 ethnic communities in Bangladesh speak more than 30 languages, most of these languages lack digital tools for translation, language identification, or preservation [4]. The Chakma language is spoken by around 600,000 to 1000,000 people, primarily in the Chittagong Hill Tracts of Bangladesh and in parts of India, including Tripura, Mizoram, and Arunachal Pradesh. The language is a vital part of the Chakma community's culture and identity [5]. Similarly, the Garo language has around 1145,323 first-language speakers, predominantly in India (Meghalaya, Assam, Tripura) and Bangladesh (northern regions). The Garo language remains an important cultural and communication tool for the Garo community [6]. The Marma language is spoken by approximately 186,700 to 220,000 first-language speakers, mostly located in the Chittagong Hill Tracts of Bangladesh. The Marma people use the language to maintain their distinct identity and heritage [7]. Some efforts have been made to address this gap, such as using pre-trained transformer models like BanglaT5 to translate Chakma to Bengali by Chakma et al. (2023)[8]. The study has shown promising results by employing techniques like back-translation and transliteration, but it also highlights how limited and fragmented current research remains. To build on this foundation, MELD introduces a multilingual dataset encompassing three major ethnic languages: Chakma [9], Garo [10], and Marma [11], alongside Standard Bengali. This dataset is intended to promote developments in low-resource language processing by allowing researchers to work on language identification, machine translation, and cultural preservation. It is written in Bengali script using transliteration ptocess. MELD provides an opportunity to bridge the gap in digital representation for ethnic languages in computational linguistics.

## 3 Data Description

**Fig. 1:**
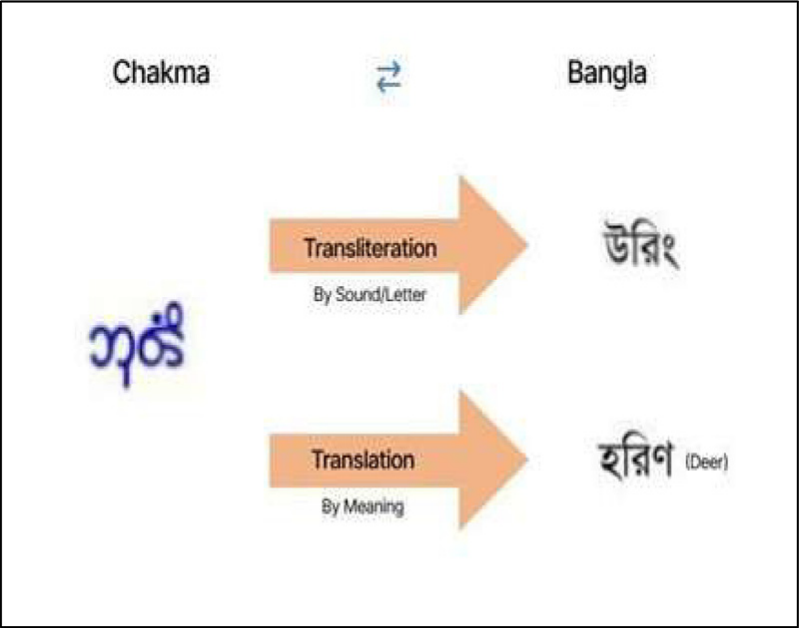
Transliteration process.

We have made our datasets openly accessible on a data repository [12]. The dataset includes linguistic samples across four categories: Chakma, Garo, Marma, and Standard Bengali. For consistency, all sentences were transliterated, meaning the Chakma, Garo, and Marma texts were written using Bengali script to represent their pronunciation. Here, transliteration refers to the phonetic representation of Chakma, Garo, and Marma languages using the Bengali script, as commonly practiced by native speakers shown in Fig. 1. We also provided translations of each sentence in both Standard Bengali and English to capture their meaning. Here, translation refers to the sentence-level meaning-based equivalents provided in Standard Bengali and English.

There are 3046 sentences in the dataset, 808 of which are in Chakma, 314 in Garo, and 292 in Marma, consisting of a direct translation in Bengali for every sentence. The structure of the dataset is illustrated in Table 1.

**Fig. 2:**
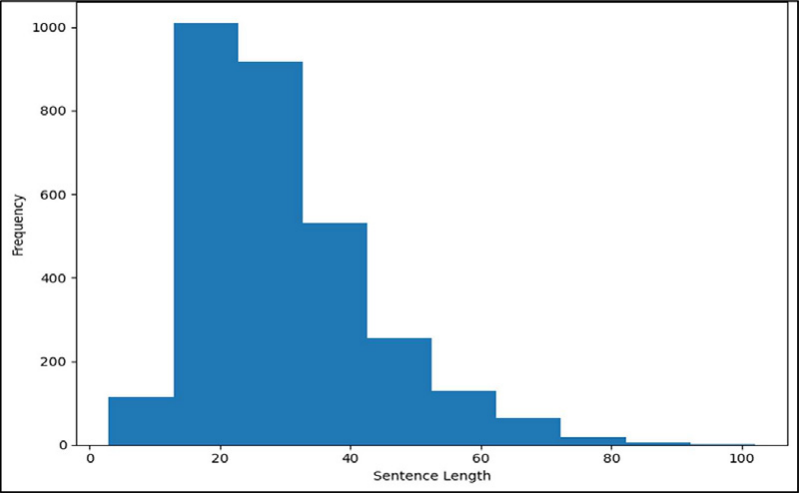
Distribution of sentence lengths.

Selecting text data that closely resembles real-world human interactions, dialogues, and discussions has been given top focus. We were able to produce a dataset that accurately reflects the terms used in regular conversations by employing this technique (Table 2) and Fig. 2 demonstrates the length of all the sentences in the dataset.

**Table 1:** Dataset description.

Dataset	No of Sample	Word Count	Maximum length (in words)	Minimum length (in words)	Average length (in words)	Maximum length (in characters)	Minimum length (in characters	Average length (in characters)
English	816	4883	16	2	5.984069	102	7	30.781863
Standard Bangla	816	4380	12	2	5.367647	83	9	30.859069
Chakma	808	4529	14	2	5.605198	83	6	28.757426
Garo	314	1680	14	1	5.350318	72	3	31.324841
Marma	292	1244	9	2	4.260274	46	7	21.058219
**Total**	3046	16,716

**Table 2:** The data are stored in a CSV file with the following columns:.

Sentence length in the dataset.

We analyzed the dataset to identify the most common words in each ethnic language by calculating word frequency within sentences. The Table 3 presents the most frequently occurring words for each language in the dataset.

**Table 3:** Most common word in dataset.

**Fig. 3:**
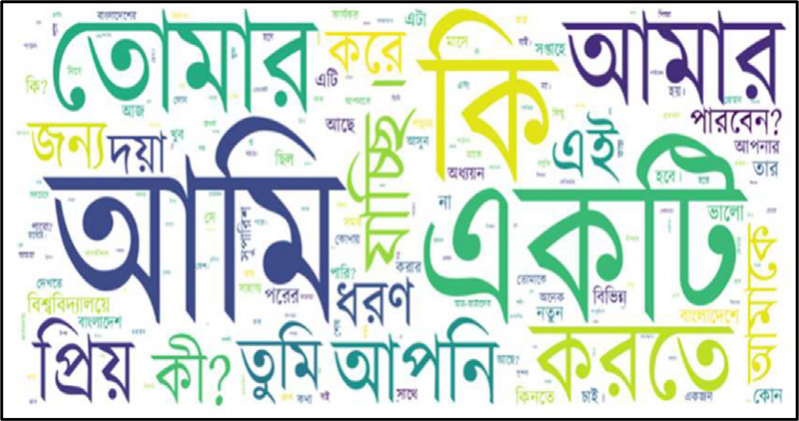
Word cloud for standard Bangla class.

**Fig. 4:**
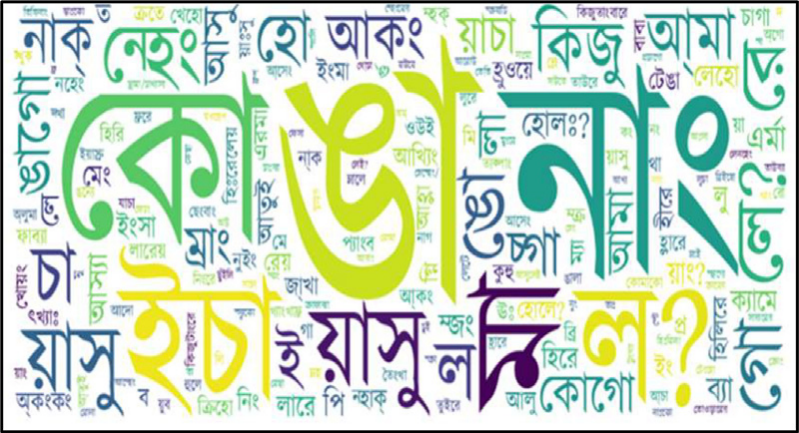
Word cloud for Marma class.

**Fig. 5:**
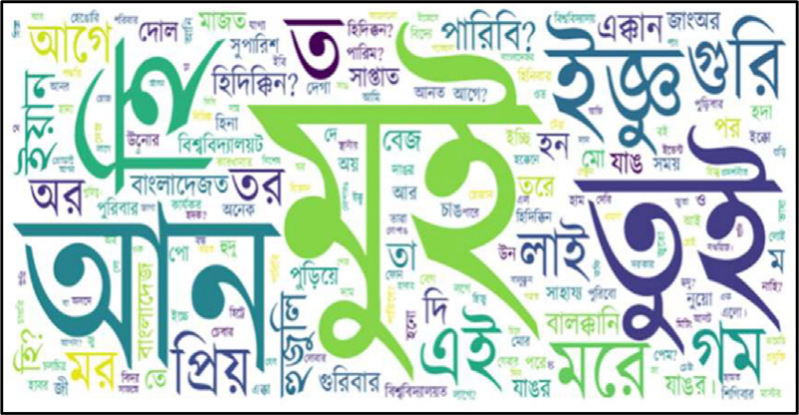
Word cloud for Chakma class.

**Fig. 6:**
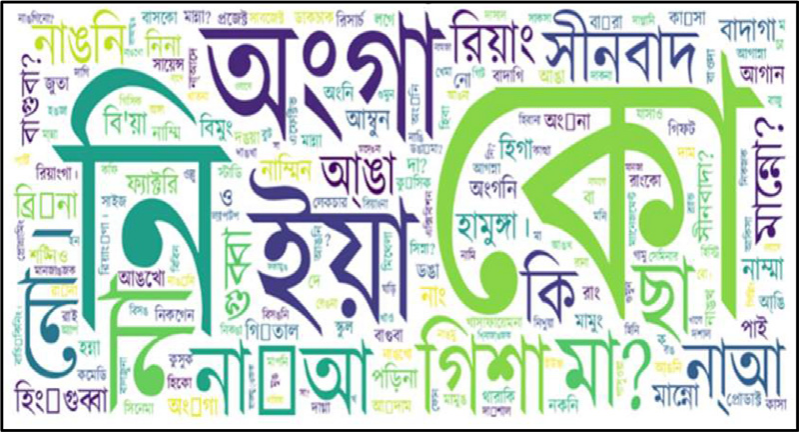
Word cloud for Garo cla.

**Fig. 7:**
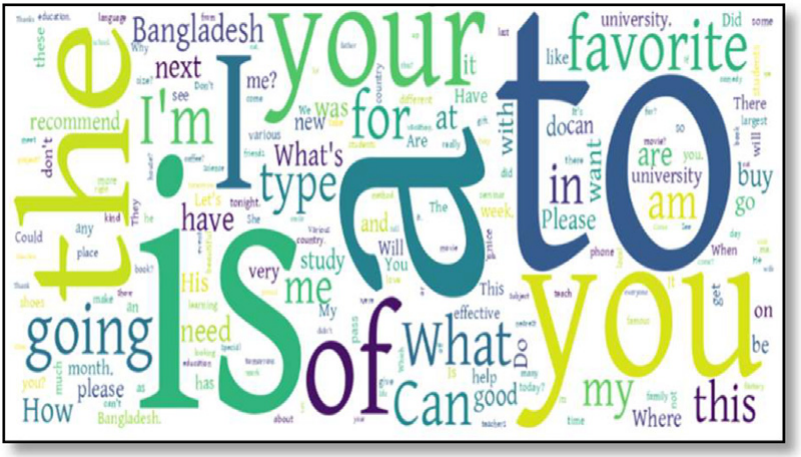
Word cloud for english.

Based on Table 3, which lists the most common words in each language within the ethnic dataset, we generated word clouds to visually represent the frequency of these words. Fig. 3, Fig. 4, Fig. 5, Fig. 6, Fig. 7 illustrate the word cloud for each language. The word clouds were generated by grouping sentences by label and concatenating them into one text per label (str.cat() in pandas). Common stop words and punctuation were removed. We used Python's WordCloud library to visualize the most frequent words without setting a manual frequency threshold. These word clouds highlight dominant vocabulary within each label, reflecting recurring themes and patterns in daily conversations. Frequent terms indicate key topics and offer insights into the linguistic focus of each category.

Although there is a lack of significant work on ethnic language processing, a few studies have provided concepts and encouragement for future research in this area. The dataset most comparable to ours is presented in Table 4.

**Table 4:** Comparison with available datasets of ethnic language. Here, the proposed dataset is significantly larger than existing comparable datasets and, notably, consists of complete sentences rather than isolated words or phrases. Furthermore, it encompasses multiple ethnic languages which are absent in currently available datasets.

	Classes	Linguistics	Dataset type	Dataset Size	Methodology (Data collection source)	Sentence	Dialect Coverage
Presented dataset[12]	5	Chakma, Marma, Garo, English, Standard_Bangla,	Text	3046	Interviews and Questionnaire	✔	X
Podder et al. [13]	2	Chakma,Bangla	Image	47	Mobile application	X (characters)	X
Ashaduzzaman and Rashel [4]	1	Marma	Text	X (not mentioned)	Books and Documents	✔	X
Rahman et al. [14]	1	Chakma	Text	1157	Interviews	✔	X

## 4 Experimental Design, Materials and Methods

### 4.1 Methodology

**Fig. 8:**
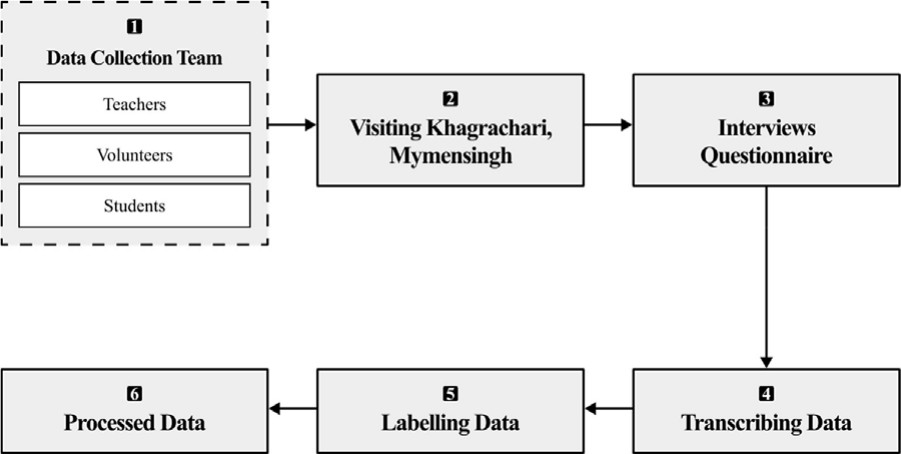
**Data collection process**. (1) Forming a team comprising volunteers, teachers, and students from the Multidisciplinary Action Research Lab; (2) Visiting the native regions of each language group; (3) Conducting interviews with local native speakers; (4) Transcribing the data with the help of volunteers who are native speakers themselves; (5) Organizing the collected samples, each with corresponding Bengali translations; Labeling the data with the assistance of multiple verified speakers of each language; and (6) Processing the dataset by adding English translations.

In Fig. 8, we present our methodology outlining the systematic steps taken to develop the dataset, ensuring the accuracy of our study.

[1] **Participants**: Seven ethnic university students fluent in their respective native languages volunteered to contribute to the dataset for the advancement of their own mother tongue. Among them, three identified Chakma as their first language, two identified Marma, and the remaining two identified Garo. The volunteers aged between 20 and 26 years, comprising five males and two females. The participants represented three indigenous groups: Chakma and Marma from Khagrachari, and Garo from Mymensingh.Given their familiarity with pronunciation and their experience in writing their native languages using the transliteration process, they were entrusted with transcribing data samples directly from interviews conducted with native speakers from the respective districts.

[2] **Tracking location**: To ensure authentic data, we visited the hometowns of each ethnic native speaker who volunteered for the study.

[3] **Data Collection**:Data samples were collected through structured interviews and written contributions from individuals aged between 20 and 78. Each sentence in the dataset reflects everyday language use, capturing day-to-day conversations and expressions common within the respective communities. During the interviews, the volunteers served as translators, facilitating communication and transcribing the responses from native speakers into written form. Standard Bengali texts were also curated to ensure dataset balance. Subsequently, English translations were added for broader usability and the dataset was expanded size to 3046 sentences from 2230 after the inclusion of Enlish translation..

[4] **Transliteration**: All data were manually transliterated into Bengali script to ensure consistency and usability by the volunteers of each language.

[5] **Annotation**: Each sample was annotated with its language category and context with at least two native speakers of each language.

[6] **Validation**: A subset of the data underwent verification by other native speakers whom we visted. Discrepancies were resolved through peer reviews and consensus-building among native-speaking university volunteers. Each sample was cross-verified by at least two speakers of the respective language to ensure consistency and minimize transliteration errors.

**Fig. 9:**
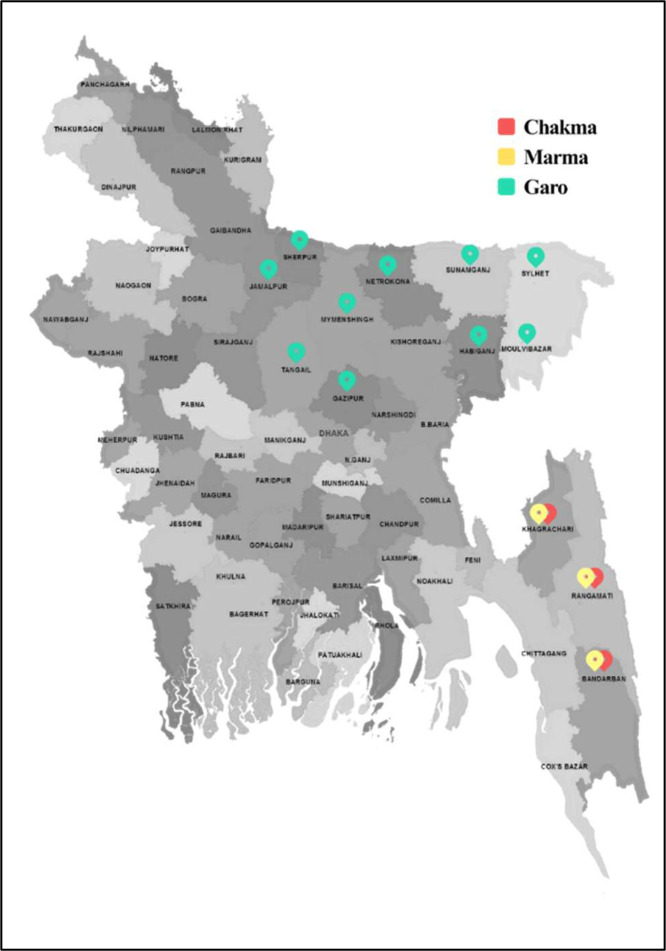
Geo location of selected ethnic groups.

**Fig. 10:**
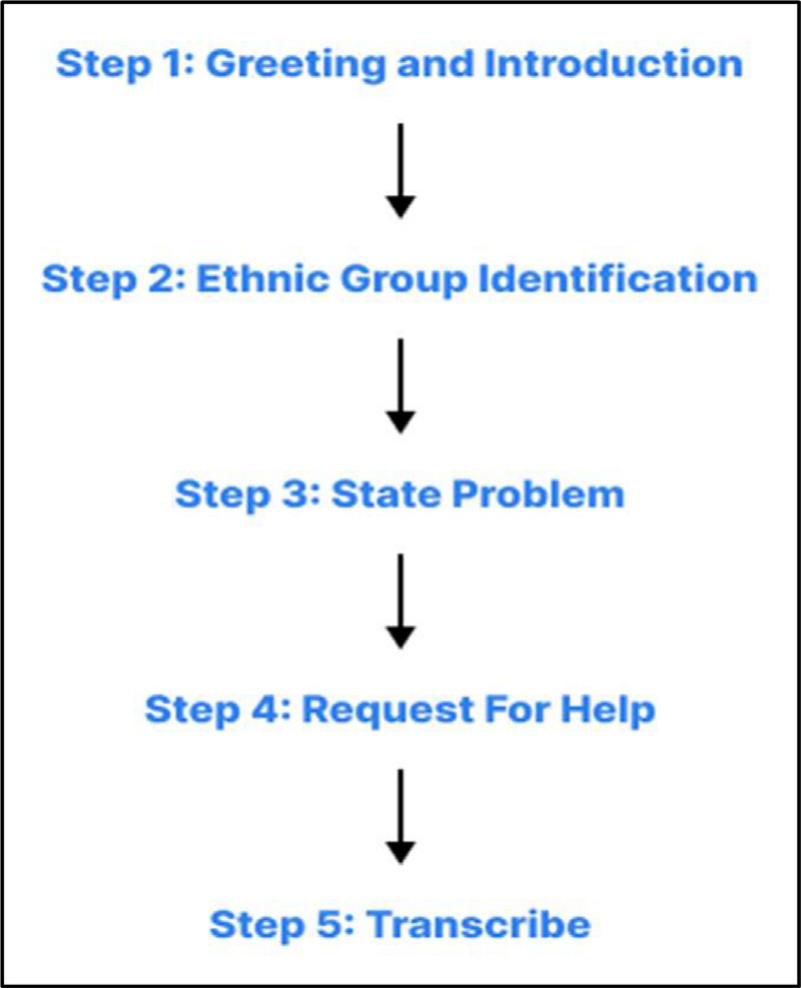
Process of interview.

The geo locations of the ethnic community of which 3 leading ethnic language that we chose are marked in Fig. 9. The process of interviewing is presented in Fig. 10.

To make sure the dataset is meticulously verified and arranged, we followed each methodical process. We addressed crucial phases including handling, labeling and confirming to preserve high quality and uniformity.

As described in Fig. 10, we conducted interviews using themes such as daily conversations, cultural expressions, greetings, and everyday interactions. We asked participants open-ended questions like:

• “How do you say ‘What are you doing?' in your language?”
• “What do you usually say when meeting a friend?”
• “Can you share a common household phrase or greeting?”

Participants responded in their native languages, and we carefully noted down their exact sentences for inclusion in our dataset. This approach ensured the collection of authentic, naturally occurring language examples.

To evaluate the effectiveness of the MELD dataset, four widely utilized supervised classification models—Naive Bayes (NB), Logistic Regression (LR), and Random Forest (RF), Decision Tree (DT)—were employed. The models were trained and tested individually on the dataset, achieving encouraging results with accuracy scores of 90.33 %, 93.44 %, 92.79 % and 89.02 % respectively. These initial outcomes demonstrate the potential of the dataset for ethnic language classification tasks. Table 5 presents the performance metrics of these models when applied to the MELD dataset. As part of future work, we aim to expand the dataset by incorporating a larger volume of samples from diverse regions and speakers, which will enhance model robustness and improve generalizability across a broader linguistic spectrum. All supplementary materials, including tables and figures, are accessible at the following github link: https://github.com/AnzirRahman/MELD.

**Table 5:** This table presents the model performance comparison of four machine learning models.

Model	Accuracy	Precision	Recall	F1-score
Naive Bayes	0.9033	0.9120	0.9033	0.8929
Logistic Regression	0.9344	0.9356	0.9344	0.9328
Random Forest	0.9279	0.9390	0.9279	0.9307
Decision Tree	0.8902	0.9060	0.8902	0.8940

These descriptions help clarify the significance of each metric in evaluating model performance.

**Accuracy:** A model’s accuracy is determined by the percentage of correct predictions it generates. It is calculated by considering both true positives and true negatives among all observations.

**Precision:** Precision refers to the percentage of correct positive predictions among all the model’s positive predictions. It focuses on how accurate the positive predictions are.

**Recall:** Recall, also known as sensitivity, represents the percentage of actual positive samples that the model correctly identified. It measures the model’s ability to recognize all relevant positive cases.

**F1 Score:** The F1 score is the weighted average of precision and recall, providing a balanced measure that considers both false positives and false negatives. It is often more informative than accuracy alone.

**Data Availability**

The dataset is available at MELD: A Multilingual Ethnic Dataset of Chakma, Garo, and Marma in Bengali Script with English and Standard Bengali translation.

## Limitations

The limitations of the MELD dataset are as follows:

• The maximum sentence length in the dataset is 14 words, which might limit the ability to capture long-range dependencies in the language.
• The total number of samples in the dataset is relatively low, which could affect the performance of models trained on this data.
• The dataset is limited to three ethnic languages: Chakma, Garo, Marma, and Standard Bengali, which restricts its applicability to other ethnic languages.
• The dataset is imbalanced in terms of class distribution, with Chakma containing more samples than Garo and Marma, which may lead to biased model performance and underrepresentation of certain language classes.
• The dataset may be subject to sampling bias, as the current dataset relies on contributions from seven university students who acted as both translators and transcribers. While they are fluent in their native languages, we acknowledge the limitation in regional diversity and the non-randomized nature of the sample.
• The sociolinguistic context, such as code-switching patterns and language usage in different domains (e.g., formal vs. informal), is not captured, which could limit the dataset’s applicability in broader linguistic and NLP analyses.

## Ethics Statement

The authors confirm that there are no ethical concerns associated with the development of this dataset. The dataset is fully anonymized, and data redistribution policies have been strictly followed. Contributors were recruited through a public notice issued by the Multidisciplinary Action Research Lab DIU, to which they voluntarily responded. All contributors were informed that their participation was voluntary with no academic credit, and informed consent was obtained prior to data collection. No incentives were provided to the volunteers. This study was conducted following the ethical guidelines of Daffodil International University Research Ethics Community(REC), and the research protocol was approved under Ethics Protocol Number *Re* f: DIU/Dean/FSIT/2025–0021

## Declaration of Generative AI and AI-assisted Technologies in the Writing Process

During the preparation of this manuscript, the authors used ChatGPT for manuscript’s language improvement. After using this tool/service, the authors reviewed and edited the content as needed and take full responsibility for the content of the publication.

## CRediT authorship contribution statement

**Mehraj Hossain Mahi:** Methodology, Data curation, Validation. **Anzir Rahman Khan:** Visualization, Writing – original draft. **Mobashsher Hasan Anik:** Data curation, Validation. **Sheak Rashed Haider Noori:** Conceptualization. **Arif Mahmud:** Writing – review & editing. **Mayen Uddin Mojumdar:** Supervision, Writing – review & editing, Conceptualization.

## Data Availability

Mendeley DataMELD: A Multilingual Ethnic Dataset of Chakma, Garo, and Marma in Bengali Script with English and Standard Bengali translation (Original data).
